# 5ʹ-Ectonucleotidase CD73/NT5E supports EGFR-mediated invasion of HPV-negative head and neck carcinoma cells

**DOI:** 10.1186/s12929-023-00968-6

**Published:** 2023-08-24

**Authors:** Enxian Shi, Zhengquan Wu, Birnur Sinem Karaoglan, Sabina Schwenk-Zieger, Gisela Kranz, Nilofer Abdul Razak, Christoph A. Reichel, Martin Canis, Philipp Baumeister, Reinhard Zeidler, Olivier Gires

**Affiliations:** 1 Department of Otorhinolaryngology, Head and Neck Surgery, LMU University Hospital, LMU Munich, Munich, Germany; 2 Institute of Structural Biology, Research Unit Therapeutic Antibodies, Helmholtz Munich, Feodor-Lynen-Str. 21, 81377 Munich, Germany

**Keywords:** CD73, EGFR, EMT, Invasion, Antagonistic antibody, HNSCC

## Abstract

**Background:**

Epithelial-to-mesenchymal transition (EMT) of malignant cells is a driving force of disease progression in human papillomavirus-negative (HPV-negative) head and neck squamous cell carcinomas (HNSCC). Sustained hyper-activation of epidermal growth factor receptor (EGFR) induces an invasion-promoting subtype of EMT (EGFR-EMT) characterized by a gene signature (“‘EGFR-EMT_Signature’”) comprising 5´-ectonucleotidase CD73. Generally, CD73 promotes immune evasion via adenosine (ADO) formation and associates with EMT and metastases. However, CD73 regulation through EGFR signaling remains under-explored and targeting options are amiss.

**Methods:**

CD73 functions in EGFR-mediated tumor cell dissemination were addressed in 2D and 3D cellular models of migration and invasion. The novel antagonizing antibody 22E6 and therapeutic antibody Cetuximab served as inhibitors of CD73 and EGFR, respectively, in combinatorial treatment. Specificity for CD73 and its role as effector or regulator of EGFR-EMT were assessed upon CD73 knock-down and over-expression. CD73 correlation to tumor budding was studied in an in-house primary HNSCC cohort. Expression correlations, and prognostic and predictive values were analyzed using machine learning-based algorithms and Kaplan–Meier survival curves in single cell and bulk RNA sequencing datasets.

**Results:**

CD73/NT5E is induced by the EGF/EGFR-EMT-axis and blocked by Cetuximab and MEK inhibitor. Inhibition of CD73 with the novel antagonizing antibody 22E6 specifically repressed EGFR-dependent migration and invasion of HNSCC cells in 2D. Cetuximab and 22E6 alone reduced local invasion in a 3D-model. Interestingly, combining inefficient low-dose concentrations of Cetuximab and 22E6 revealed highly potent in invasion inhibition, substantially reducing the functional IC_50_ of Cetuximab regarding local invasion. A role for CD73 as an effector of EGFR-EMT in local invasion was further supported by knock-down and over-expression experiments in vitro and by high expression in malignant cells budding from primary tumors. CD73 expression correlated with EGFR pathway activity, EMT, and partial EMT (p-EMT) in malignant single HNSCC cells and in large patient cohorts. Contrary to published data, CD73 was not a prognostic marker of overall survival (OS) in the TCGA-HNSCC cohort when patients were stratified for HPV-status. However, CD73 prognosticated OS of oral cavity carcinomas. Furthermore, CD73 expression levels correlated with response to Cetuximab in HPV-negative advanced, metastasized HNSCC patients.

**Conclusions:**

In sum, CD73 is an effector of EGF/EGFR-mediated local invasion and a potential therapeutic target and candidate predictive marker for advanced HPV-negative HNSCC.

**Supplementary Information:**

The online version contains supplementary material available at 10.1186/s12929-023-00968-6.

## Background

Head and neck squamous cell carcinomas (HNSCC) encompass a group of solid cancers of different localizations within the upper aerodigestive tract exhibiting varying molecular compositions and clinical outcomes [27]. Advanced HNSCC commonly associate with the presence of lymph-node metastases (LN-mets) at initial diagnosis and with the rapid appearance of local, loco-regional, and/or distant recurrences [54]. Advanced stage disease accounts for approximately 50% of cases, and results in a dismal 5-year overall survival (OS) below 35% [46], ultimately representing a considerable medical challenge. HNSCC progression is attributed to field carcinogenesis and to their propensity to disseminate in conjunction with high level of resistance to multimodal aggressive regimens comprising surgical removal and adjuvant radio(chemo)therapy and/or immunotherapy. LR are defined as tumors that occur within less than three years and within few centimeters of the initial tumor in the field of resection and/or high-dose irradiation. LR are observed in 10–30% of cases despite histologically tumor-free resection margins (R0 resection), implying the presence of minimal or molecular residual disease (MRD) despite R0 resection [3, 41]. The formation of metastases and consequently of an antecedent recurrence-inducing tumor budding were connected to an epigenetically orchestrated trans-differentiation program termed epithelial-to-mesenchymal transition (EMT) in malignant HNSCC cells [15, 16, 22, 43, 44, 48].

Various forms and expression degree of EMT have been described that contribute to central aspects of cancer progression including enhanced migration and invasion, resistance to treatment, and cell stemness [7, 24]. Puram et al*.* reported a partial EMT (p-EMT) induction based on single cell RNA sequencing (scRNAseq) of oral cavity cancers and observed a preferential localization of malignant p-EMT cells at the edges of tumor areas [44]. Loss of epithelial marker EpCAM and concurrent expression of mesenchymal marker Vimentin on the tumor margins were corroborated [4] and the degree of p-EMT prognosticated the clinical outcome in HNSCC [47]. Mechanistically, p-EMT was shown to be triggered by stroma-resident cancer-associated fibroblasts (CAFs) through release of TGBβ and the activation of TGBβ receptors [44]. Additionally, induction of EMT in HNSCC via sustained hyper-activation of the epidermal growth factor receptor (EGFR) and the MAPK pathway was described by us and others [11, 13, 39]. Subsequently, we identified an ‘EGFR-EMT_Signature’ composed of 181 genes, from which a 5-gene signature was extracted that predicted OS of HNSCC patients [48].

The discovery that various forms of EMT including EGFR-EMT are relevant drivers of HNSCC progression provides novel prognostic, predictive, and therapeutic options in form of gene signatures associated with various EMT states of malignant cells. For example, integrin beta 4 (ITGB4), which is a constituent of the EGFR-EMT 5-gene prognostic signature, was identified as a potential candidate for blocking antibodies and as predictive marker for the response of advanced, metastasized HNSCC patients to Cetuximab [48]. In the present study, we focused on the 5ʹ-nucleotidase CD73/NT5E that is comprised in the EGFR-EMT signature [48]. CD73 is a membrane-tethered 5´-ectoenzyme hydrolyzing adenosine monophosphate (AMP) to adenosine (ADO), which is immunosuppressive at high concentrations [61]. Consequently, CD73 is considered a novel immune checkpoint molecule and an attractive druggable target for blocking monoclonal antibodies [2, 58]. Adenosinergic pathways have been linked to EMT regulation [17, 18] and CD73 has EMT-related functions beyond ADO production, *i.e.* as a receptor for components of the extracellular matrix (ECM) that promotes cell migration [18]. Correlations of CD73 expression with gene signatures of EMT and metastasis in malignant single HNSCC cells and, following deconvolution of bulk-seq data, with cancer-associated fibroblasts (CAFs) were reported [9, 49]. Furthermore, an interplay between CD73 and EGFR has been described in HNSCC, in which CD73 supported EGF/EGFR signaling [45]. However, an induction of CD73 via EGFR and CD73´s role(s) in EGFR-EMT-dependent functions in HPV-negative HNSCC remain unexplored. Despite publications on a correlation of CD73 expression with the clinical endpoints OS, disease-free and disease-specific survival, a prognostic value of CD73 in HNSCC remains unclear due to a common lack of HPV-stratification of cohorts in published data [9, 36, 45, 64]. Therefore, we assessed CD73 as a potential target to inhibit EGFR-EMT-related tumor cell invasion, as a prognostic parameter, and as a predictive marker of response to Cetuximab treatment.

## Methods

### Cell lines and treatments

Human hypopharyngeal and esophageal cell lines FaDu and Kyse30 (ATCC, Manassas, VA, USA) were regularly confirmed via STR typing. Cells were cultured and passaged in high glucose (4.5 g/mL) RPMI or DMEM with 10% FCS and 1% penicillin/streptomycin, 5% CO2 atmosphere at 37 °C. For treatment purposes, cells were seeded in 6-well plates or 96-well low-adhesion plates when forming spheroids. Treatment with EGF^low^ and EGF^high^ (10 ng/mL and 50-75 ng/mL, respectively; PromoCell PromoKine, Heidelberg, Germany), Cetuximab (10–20 µg/mL, Erbitux, Merck Serono, Darmstadt, Germany), anti-CD73 antibody 22E6 (2.5 µg/mL, and 5 µg/mL [21]), APCP (Adenosine 5′-(α,β-methylene)diphosphate, Sigma Aldrich, Burghausen, Germany), MEK inhibitor AZD6244 (0.1 µM, Selleckchem) and AKT inhibitor MK2206 (0.1 µM, Selleckchem) were conducted under serum-free conditions to avoid a potential impact of proliferation on functional assays. Mitomycin C (Bioreagent, Schwerte, Germany) was further used at the I_C50_ of 0.02 µg/mL for 72 h during 3D invasion in FaDu cells. In 3D invasion assays, hybridoma supernatant from the cognate IgG2α isotype (2.5, 5 µg/mL [21]) and goat anti human IgG (1 µg/ml, Jackson Immuno Research) served as controls.

FaDu wild-type cells were transfected with CD73-specific shRNA expression plasmids (pRP[shRNA]-EGFR:P2A:Puro-U6 > shRNA targeting hNT5E (5’-TAAGTTTACGTGTCCAAATTT-3’) and pRP[shRNA]-EGFR:P2A:Puro-U6 > irrelevant scramble shRNA (5’-CCTAAGGTTAAGTCGCCCTCG-3’) as control), sorted for GFP-positivity and cultured in the presence of 2 µg/ml puromycin. CD73 over- and re-expressed was achieved with a pRP[Exp]-Hygro-CAG > hNT5E plasmid in stable bulk transfectants. All plasmids were sourced from VectorBuilder (Neu-Isenburg, Germany) and transfected with Lipofectamine 3000 transfection reagent as recommended by the manufacturer (Thermo Fisher Scientifc, Germering, Germany).

### Flow cytometry

After seeding and culturing FaDu and Kyse30 cells in 6-well plates at 250,000 cells/well for 24 h, cells were starved in serum-free RPMI or DMEM with 1% penicillin/streptomycin in 5% CO2 atmosphere at 37 °C for 16 h. Following starvation, cells were subjected to the indicated treatments for 72 h, and collected for staining. Cells were stained with CD73-specific Alexa Fluor® 647-conjugated antibody, primary CD73-specific antibody 22E6 (1:100 in PBS-3% FCS, 60 min at 4 °C, [21]) or rat IgG2α isotype control antibody (sc-2026, Santa Cruz Biotechnology, 1:100 in PBS 3% FCS, 60 min at 4 °C). After washing three times in PBS-3% FCS, cells of the isotype control groups and stained groups were stained with Alexa Fluor® 647-conjugated secondary antibody (1:100, 60 min at 4 °C, Jackson Immuno Research) or Alexa Fluor® 594-conjugated secondary antibody (1:100, 60 min at 4 °C, A11007, Molecular Probes).

### Cytotoxicity assay

CD73-specific antibody 22E6 and Mitomycin C were tested for cellular cytotoxicity at different concentrations. FaDu and Kyse30 cells were seeded at 3,000 cells/well in 96-well plate and left overnight to fully adhere. Cells were treated with 22E6, Mitomycin C, or left untreated as control and incubated for 24 h and 72 h. At the indicated time points, the Cell Counting Kit-8 (Dojindo, Kumamoto, Japan) was used according to the manufacturer's protocol. Absorbance at 450 nm was measured via a colorimeter (VersaMax Microplate Reader, Molecular Devices, San Jose, CA, USA) to determine cell viability.

### 2D migration and invasion assay

2D migration and invasion assay were performed in transwell Boyden chambers (8.0 µm, Merck Millipore Ltd., Germany). Boyden chambers were precoated with Matrigel for invasion assay (1 mg/mL, Corning, Germany) and were left untreated for migration assay. After serum-free starvation for 16 h, cells were seeded at 2 × 10^5^ cells in 300 µl serum-free medium into the upper inserts, while lower chambers contained different treatments. After 24 h of migration or invasion, cells were stained with crystal violet for 20 min. After swiping off cells attached on the top of the insert, migrated and invaded cells in different groups were imaged with a light microscope (Olympus BX43). Quantification of migrated and invaded cells was performed with the QCM™ 24-Well Colorimetric Cell Migration Assay Kit (Merck Millipore Ltd., Germany) via a colorimeter (VersaMax Microplate Reader, Molecular Devices, San Jose, CA, USA).

### Spheroid formation and 3D invasion assay

FaDu cells were seeded at 3,000 cells/well in 96-well low-adhesion plates and spheroids were allowed to form in high glucose (4.5 g/mL) DMEM 10% FCS, 1% penicillin/streptomycin, 5% CO2 atmosphere at 37 °C for 72 h. After spheroid formation, glass bottom dishes (35 mm, Ibidi, Germany) were coated with 200 µl Matrigel (3 mg/mL, Corning, Germany) containing 6–8 spheroids/well. Embedded spheroids were incubated for 1 h at 5% CO2 atmosphere at 37 °C in the absence of growth factors. After Matrigel polymerization, each dish was treated with serum-free DMEM containing the indicated treatment. After 72 h, spheroids were imaged on a DMi8 microscope (Leica, Nussloch, Germany). Fiji Image J2 was applied to quantify invasive area (total area minus central spheroid area) and invasive distance (average distance of the furthest 15–20 cells from the spheroid center).

### Human samples, immunohistochemistry, tumor budding analysis and scoring

Clinical samples were taken as 8 mm punch biopsies of resected primary tumors and normal mucosa beyond resection margins and were cryo-preserved by snap-freezing in tissue-Tek® (Sakura, Finetek, The Netherlands). For staining, samples were processed to 5 µm sections. CD73 antibody 22E6 (1:100) was used for immunohistochemistry (IHC) staining with the avidin–biotin-peroxidase complex method (Vectastain, Vector laboratories, Burlingame, CA, US). IHC staining pictures were recorded by light microscopy (Olympus BX43, Munich, Germany). IHC scoring was evaluated by two experienced scorers independently in a blinded manner as described before [34]. Briefly, antigen expression was scored on a scale of 0–3 (0: no expression, 1: weak; 2: intermediate; 3: strong) and as a proportion of cells in each expression value in percent. The IHC score represents antigen intensity multiplied by proportion (IHC score 0–300). Tumor budding, defined as the presence of single tumor cells or small clusters of less than five tumor cells at the invasive tumor areas, was evaluated as negative and positive by two experienced scientists independently in a blinded manner.

### Dataset source and preprocessing

Normalized gene expression data and clinical information of the TCGA-HNSCC cohort were downloaded from the cBioPortal website (https://www.cbioportal.org/). Two microarray datasets of HNSCC, GSE65858 and Fred Hutchinson Cancer Research Center (FHCRC) cohort (GSE41613), were retrieved from Gene Expression Omnibus (GEO). All gene expression values were log2-transformed prior to further analysis. Only patients with HPV-negative HNSCC were selected for subsequent analysis, including n = 415 (TCGA), n = 97 (FHCRC) and n = 196 (GSE65858) samples, respectively. Two datasets of cetuximab treated HNSCC patients (GSE65021 and GSE84713) were downloaded from GEO. A pre-processed HNSCC single-cell RNA-sequencing dataset (GSE103322) was downloaded from GEO and imported into R ‘Seurat’ package for further analysis. The top 10 samples with the highest number of cancer cells were retained for further analysis as described previously [44].

### Gene set variation analysis (GSVA)

EGFR pathway activity and Hallmark EMT gene signatures were obtained from Molecular Signatures Database (MSigDB) (https://www.gsea-msigdb.org/gsea/msigdb/). Partial EMT gene and EGFR-mediated EMT gene signatures were derived from previously published studies [44, 48]. GSVA scores of individual gene signatures for each sample in bulk-seq or each cancer cell in scRNA-seq were calculated using R 'gsva'. Correlations between CD73 expression and GSVA score was calculated and visualized using R ‘corrgram’.

### Statistical analysis

Data were evaluated by one-way ANOVA analysis followed by Tukey’s multiple comparison test or by t-test (GraphPad Prism version 9.0.0 for Windows, GraphPad Software, San Diego, California USA). All values are given as mean ± S.D. with n values, and throughout the study, p values ≤ 0.05 are considered significant. Survival differences between groups (divided by median of CD73 expression) were assessed using the Kaplan–Meier method and log-rank test. Area under the curve (AUC) was calculated and visualized using R ‘pROC’. The relationship between expression of CD73 and clinical parameters in the LMU cohort was analyzed by Fisher´s exact test. Multivariate logistic regression was used to determine relationships between expression of CD73 and time to relapse after cetuximab treatment.

## Results

### EGFR up-regulates CD73 expression via the MAPK axis

Sustained hyperactivation of EGFR with high-dose EGF (EGF^high^) results in EMT induction in Kyse30 and FaDu cells, which is dependent on MEK but not AKT activity [39]. Transcriptomic profiling of EGFR-mediated EMT revealed a gene signature of exclusively regulated n = 181 genes (DEGs; differentially regulated genes, Log2FC > 0.5, adj. p-value < 0.05), including 5´-ectonucleotidase CD73 (encoded by the *NT5E* gene) [48].

**Fig. 1:**
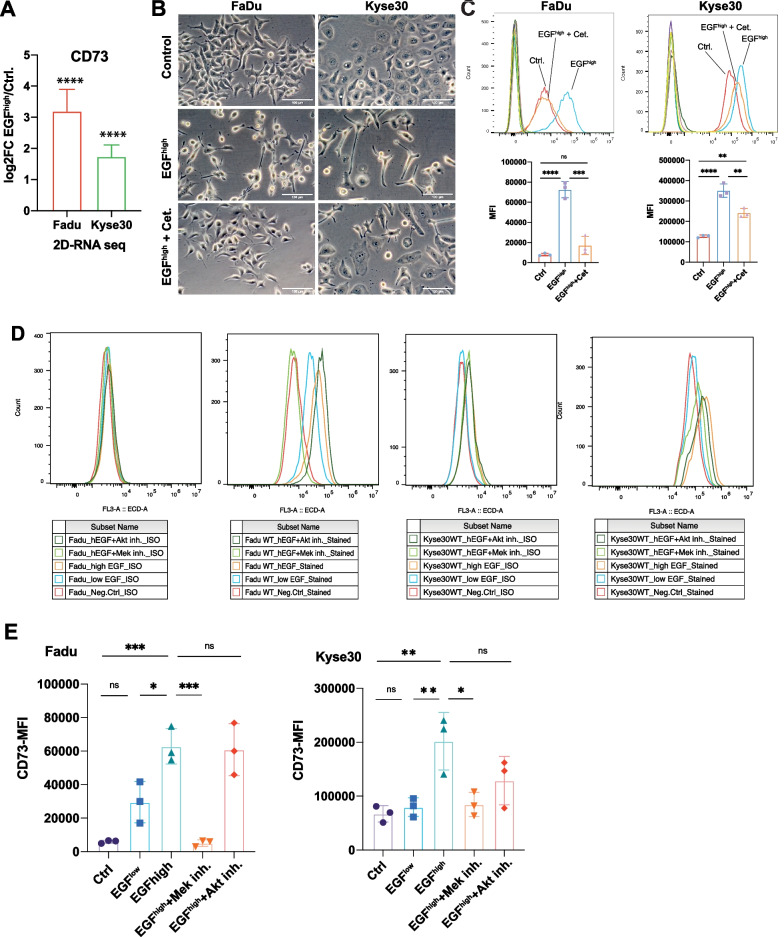
CD73 expression and regulation. **A** Relative expression of CD73 mRNA is shown as mean log2 fold change (Log2FC) of EGF-treated versus untreated samples with SEM from bulk RNAseq results following treatment of Kyse30 and FaDu cells with high-dose EGF (50 ng/mL; 72 h) (n = 4 independent experiments) [48]. **** p < 0.0001 **B** FaDu and Kyse30 cell lines were maintained under control condition (serum-free), treated with an EMT-inducing concentration of EGF (50 ng/mL; 72 h), or with a combination of EGF and Cetuximab (Cet.; 10 µg/mL; 72 h). Shown are representative bright light microscopy images of each treatment. **C** Upper: FaDu and Kyse30 cells were treated as in (B) and CD73 expression was assessed by flow cytometry with specific antibodies. CD73 histograms are marked according to their related treatments. All other histograms represent negative controls. Lower: Mean fluorescence intensities (MFI) of control treatment, EGF treatment, and EGF + Cetuximab treatment are shown as mean with SD from n = 3 independent experiments. Ns: not significant; ** p < 0.01; *** p < 0.001; **** p < 0.0001. Singular data points are depicted. **D** FaDu and Kyse30 cells were treated as described in legends with EGF^low^ (1.8 nM, 72 h), EGF^high^ (9 nM, 72 h), and EGF^high^ in combination with MEK inhibitor AZD6244 (0.1 µM) of AKT inhibitor MK2206 (0.1 µM). CD73 expression was assessed by flow cytometry with specific antibodies. Shown are representative histograms from n = 3 independent experiments. Left: isotype controls, right: specific staining. **E** Mean fluorescence intensities (MFI) of control treatment, EGF^low^, EGF^high^, and EGF^high^ plus MEK or Akt inhibitor treatment are shown as mean with SD from n = 3 independent experiments. Ns: not significant; ** p < 0.01; *** p < 0.001. Singular data points are depicted

Comparison of EGF^high^-treated FaDu and Kyse30 cells with untreated samples showed a significant Log2FC of 3.2 ± 0.69 and 1.73 ± 0.38 of CD73, respectively (Fig. 1A). Induction of EMT has been confirmed at the level of candidate genes (i.e. Slug, Snail, Zeb1) and through 3´RNA-seq [39, 48]. Induction of CD73 following EGF^high^ treatment was validated in independent experiments. EGF^high^ resulted in morphological changes associated with EMT, which were counteracted by co-treatment with the antagonizing anti-EGFR therapeutic antibody Cetuximab in FaDu and Kyse30 cells (Fig. 1B). In both cell lines, EGF^high^ treatment enhanced CD73 protein expression at the plasma membrane, which was counteracted by Cetuximab co-treatment. Mean induction levels at the plasma membrane were 9.06-fold and 2.76-fold in FaDu and Kyse30 cells, respectively (Fig. 1C), which correlated tightly with transcript changes (Fig. 1A, Log2FC 3.2 = 9.19 FC; Log2FC 1.73 = 3.32 FC).

Pathways involved in EGFR-mediated CD73 up-regulation were addressed upon treatment with low-dose EGF (EGF^low^), which fails to induce EMT, EGF^high^, and EGF^high^ in combination with MEK or AKT inhibitors. EGF^low^ did not significantly (FaDu) or not at all induce CD73 expression (Kyse30), whereas EGF^high^ potently up-regulated CD73 in both cell lines. In concordance with pathways involved in EMT induction, CD73 up-regulation by EGF^high^ depended on MEK rather than Akt. Inhibition of MEK resulted in a complete block of CD73 up-regulation (Fig. 1D, E). Hence, CD73 expression at the plasma membrane is enhanced during EGFR-EMT via MEK-dependent signaling.

### Inhibition of CD73 impacts on 2D EGFR-mediated migration and invasion

**Fig. 2:**
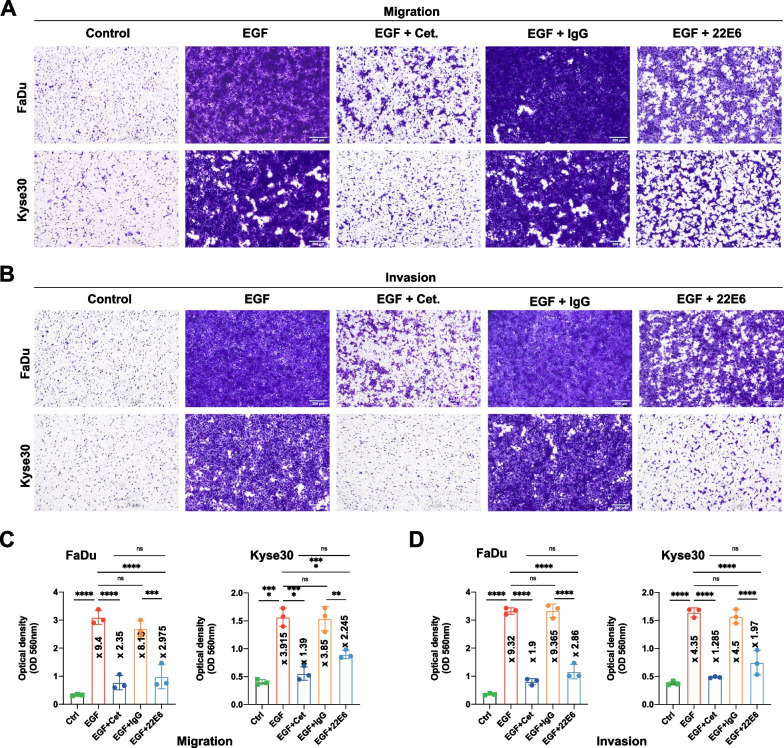
CD73 effects on migration and invasion. **A-B** Representative staining of migrated (**A**) and invaded (**B**) FaDu and Kyse30 cells in a Boyden chamber assay with the indicated treatments. EGF: 50 ng/mL; Cet.: cetuximab 10 µg/mL; IgG: immunoglobulin control; 22E6: antagonizing anti-CD73 monoclonal antibody (5 µg/mL) (n = 3 independent experiments; all treatments were performed for 24 h). **C-D** Quantitative analyses of tumor cell migration (**C**) and invasion (**D**). Shown are mean with SD of n = 3 independent experiments including singular data points and fold induction values. *Ns* not significant; ** p < 0.01; *** p < 0.001; **** p < 0.0001

CD73-antagonizing antibody 22E6 [21] was used to block the enzymatic activity of CD73 in EGFR-mediated migration and invasion of FaDu and Kyse30 cells in a Boyden chamber system. Potential cellular cytotoxicity of 22E6 was excluded in a concentration range up to 10 µg/mL (Additional file 1: Fig. S1). EGF^high^ treatment induced a pronounced migration and invasion of FaDu and Kyse30 cells, which was blocked by co-treatment with Cetuximab. 22E6 reduced EGFR-mediated migration and invasion at a concentration of 5 µg/mL, while an irrelevant isotype control antibody showed no such effects (Fig. 2A, B). Quantification of cell migration and invasion confirmed a 9.4- and 3.9-fold induction of migration and a 9.3- and 4.4-fold induction of invasion in FaDu and Kyse30 cells upon EGFR stimulation, respectively. Both, Cetuximab and 22E6 antibodies repressed migration by 75% and 68.4% in FaDu and by 64.5% and 43% in Kyse30, respectively. Invasion was suppressed by Cetuximab and 22E6 by 80% and 69.3% in FaDu and by 70.5% and 55% in Kyse30, respectively (Fig. 2C, D).

### 22E6 represses EGFR-mediated local invasion

**Fig. 3:**
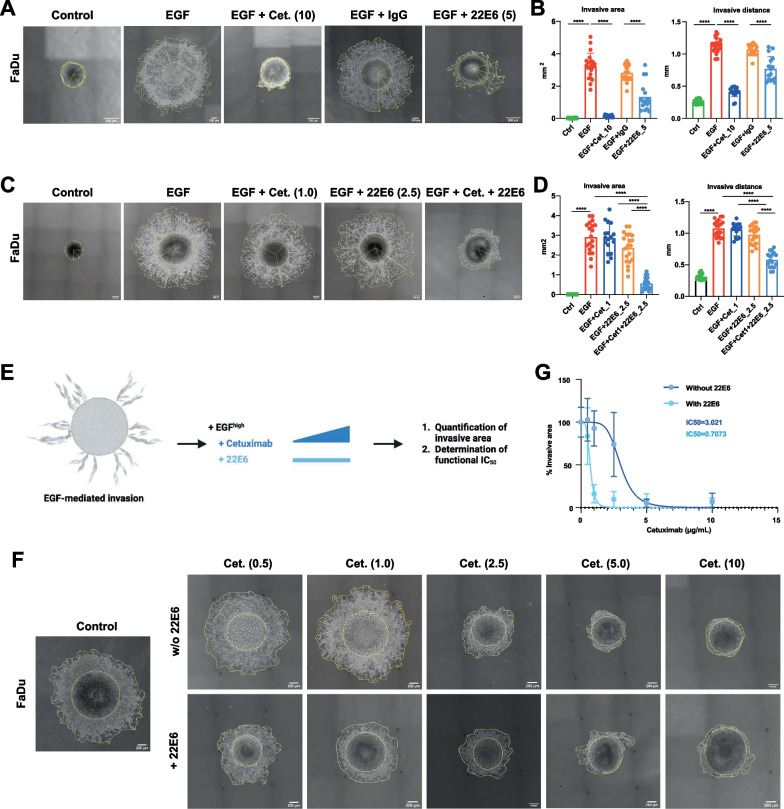
CD73 blocking inhibits EGF-induced local invasion in a 3D model. **A** FaDu cell spheroids in Matrigel were treated with the indicated compounds. EGF: 50 ng/mL; Cet.: cetuximab 10 µg/mL; IgG: immunoglobulin control 5 µg/mL; 22E6: antagonizing anti-CD73 monoclonal antibody 5 µg/mL. Yellow contours and lines served for quantification of invasive area and invasive distance, respectively. All treatment were performed for 72 h in the absence of fetal bovine serum. **B** Quantitative analysis of invasive area (left) and invasive distance (right) from n = 3 independent experiments with multiple spheroids per treatment. Singular data points are depicted. **** p < 0.0001. **C** FaDu cell spheroids in Matrigel were treated with the indicated compounds. EGF: 50 ng/mL; Cet.: cetuximab 1 µg/mL; 22E6: antagonizing anti-CD73 monoclonal antibody 2.5 µg/mL, and a combination of Cetuximab and 22E6. All antibody concentrations were equalized upon addition of immunoglobulin control antibody. Yellow contours and lines served for the quantification of invasive area and invasive distance, respectively. All treatment were performed for 72 h. **D** Quantitative analysis of invasive area (left) and invasive distance (right) from n = 3 independent experiments with multiple spheroids per treatment. Singular data points are depicted. **** p < 0.0001. **E** Schematic representation of the assessment of functional IC50 values of Cetuximab in the absence and presence of 22E6. **F** FaDu cell spheroids in Matrigel were treated with the indicated concentrations of Cetuximab in the absence or presence of 22E6 at the individually ineffective concentration o 2.5 µg/mL. Micrographs were taken at 72 h and are representative of n = 3 independent experiments with multiple spheroids. **G** Dose–response curve of the EGFR-mediated invasive area as a function of Cetuximab concentration without or with additional treatment with 22E6. Functional IC50 values of Cetuximab regarding the inhibition of EGFR-mediated invasion are indicated

Effects of CD73 inhibition on local invasion induced by EGF^high^ were addressed in a 3D spheroid model. Spheroids were formed from FaDu HNSCC cells and transferred into Matrigel as surrogate for extracellular matrix (ECM) under serum-free conditions to reduce a potential influence of growth factors. Following addition of EGF^high^, single FaDu cells detached from the spheroid structure and invaded the surrounding ECM. Co-treatment with Cetuximab strongly reduced local invasion into ECM, demonstrating the specificity for EGFR signaling. In line with results of 2D invasion, antibody 22E6 showed a repression of EGFR-mediated local invasion, whereas control IgG did not (Fig. 3A). Quantification of invasion area and invasive distance showed a significant induction of both following EGF^high^ treatment with a mean fold induction of 95.4-fold and 4.4-fold, respectively. Cetuximab reduced the invasive area by 95% and the invasive distance by 61.4%. 22E6 reduced the invasive area by 52.3% and the invasive distance by 27.9% (Fig. 3B).

Next, we assessed combinations of Cetuximab and 22E6 in the inhibition of local invasion. Low-dose concentrations of Cetuximab or 22E6 were chosen that individually had no significant effect on EGF-mediated invasion (1 µg/mL and 2.5 µg/mL, respectively). Combining these per se ineffective antibody concentrations strongly reduced the invasive area and impacted on invasive distance in a significant manner. Combination of Cetuximab and 22E6 reduced the invasion area by 80.6% and the invasive distance by 46% (Fig. 3C, D). Therefore, we determined the functional IC_50_ of Cetuximab in blocking EGF-mediated local invasion. FaDu cell spheroids were monitored following EGF^high^ treatment in combination with increasing concentrations of Cetuximab (0.5–10 µg/mL) alone or in combination with 22E6 at the ineffective concentration of 2.5 µg/mL (Fig. 3E). Cetuximab efficiently inhibited local invasion starting at a concentration of 2.5 µg/mL and showed maximal inhibition at 10 µg/mL, whereas a combinatorial treatment with 22E6 was effective already at the lowest concentration of Cetuximab of 0.5 µg/mL (Fig. 3F). Accordingly, the functional IC_50_ of Cetuximab was reduced upon co-treatment with 22E6 from 3.0 µg/mL to 0.71 µg/mL (Fig. 3G). Hence, EGFR and CD73 blockade have combinatorial effects on local invasion mediated via EGFR-EMT in HNSCC.

### CD73 is an effector, not a regulator of EGFR-EMT

**Fig. 4:**
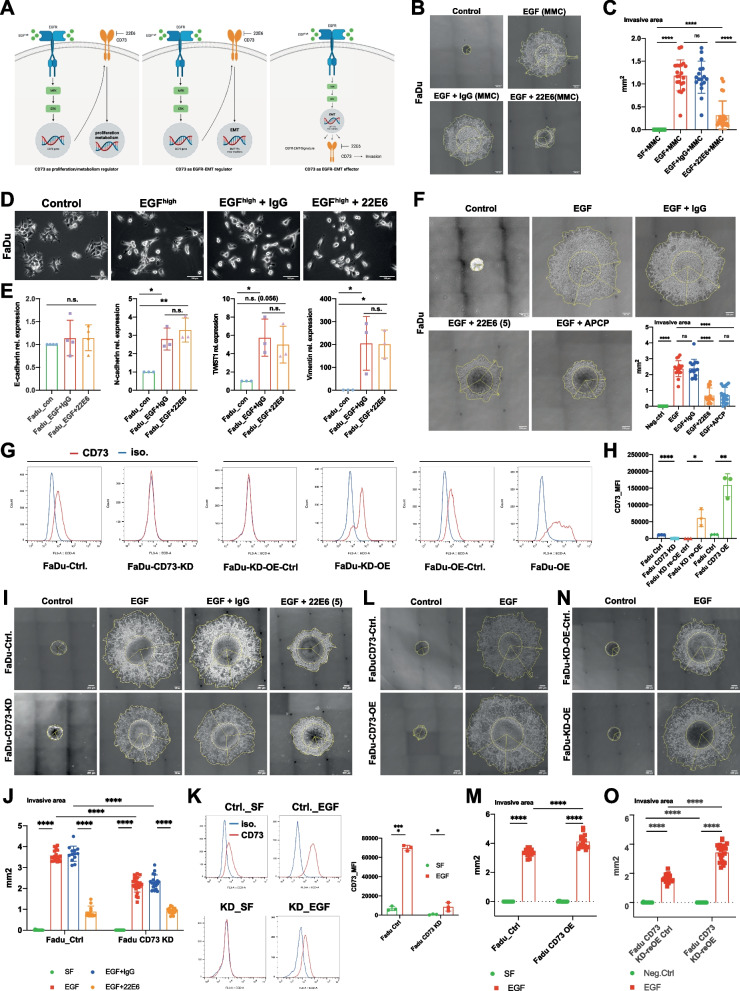
CD73 is an effector not a regulator of EGF-EMT. **A** Schematic representation of possible roles of CD73 in EGFR-EMT. CD73 may act as a regulator of proliferation/metabolism or EMT, or, alternatively, may represent an effector molecule downstream of EGFR-mediated EMT. **B** FaDu cell spheroids in Matrigel were treated mitomycin C (0.02 µg/mL) and with the indicated compounds. EGF: 50 ng/mL; IgG: immunoglobulin control 5 µg/mL; 22E6: antagonizing anti-CD73 monoclonal antibody 5 µg/mL. Yellow contours and lines served for quantification of invasive area. All treatment were performed for 72 h in the absence of fetal bovine serum. **C** Quantitative analysis of invasive area from n = 3 independent experiments with multiple spheroids per treatment. Singular data points are depicted. ns not significant, **** p < 0.0001. **D** FaDu cells were maintained under control condition (serum-free), treated with an EMT-inducing concentration of EGF (50 ng/mL; 72 h), or with a combination of EGF and immunoglobulin IgG or 22E6 (5 µg/mL; 72 h). Shown are representative bright light microscopy images of each treatment. **E** FaDu cells treated as in (C) were subjected to quantitative RT-PCR with primers specific for E-cadherin, N-cadherin, Twist1, and vimentin. Shown are mean relative mRNA expression levels normalized to control treatments from n = 3 independent experiments. ns: not significant, * p < 0.05, ** p < 0.01. **F** FaDu cell spheroids in Matrigel were treated with the indicated compounds. CD73 inhibitor APCP was used at 50 µM concentration. Quantitative analysis of invasive area from n = 3 independent experiments with multiple spheroids per treatment are shown in the lower right panel. Singular data points are depicted. ns not significant, **** p < 0.0001. **G** CD73 knock-down (KD), over-expression (OE), and CD73 re-expression in knock-down cells (KD-OE) in FaDu cells were analyzed for CD73 expression by flow cytometry with specific antibodies (CD73) or isotype control (iso.). Shown are representative histograms for each cell lines with cognate control cells. **H** Standardized mean fluorescence intensities of CD73 cell surface expression are shown as means with SD from n = 3 independent experiments. * p < 0.05, ** p < 0.01, **** p < 0.0001. **I**, **L**, **N** FaDu control (Ctrl.), CD73 knock-down (CD73-KD) (**I**), CD73 over-expression (CD73-OE) (**L**), and CD73 re-expression in knock-down cells (CD73-KD-OE) (**N**) cell spheroids in Matrigel were treated with the indicated compounds. EGF: 50 ng/mL; IgG: immunoglobulin control 5 µg/mL; 22E6: antagonizing anti-CD73 monoclonal antibody 5 µg/mL. Yellow contours and lines served for quantification of invasive area. All treatment were performed for 72 h in the absence of fetal bovine serum. **J**, **M**, **O** Quantitative analysis of invasive area from n = 3 independent experiments with multiple spheroids per treatment from spheroids shown in **I**, **L**, **N**, respectively. Singular data points are depicted. ns not significant, **** p < 0.0001. **K** Controls and CD73 knock-down (KD) FaDu cells were analyzed for CD73 expression by flow cytometry with specific antibodies (CD73) or isotype control (iso.). Cells were either kept under serum-free condition (Ctrl.-SF, KD-SF) or treated with EGF (50 ng/mL) (Ctrl.-EGF, KD-EGF). Shown are representative histograms for each cell lines with cognate control cells. Standardized mean fluorescence intensities of CD73 cell surface expression are shown as means with SD from n = 3 independent experiments. * p < 0.05, **** p < 0.0001

It is thus conceivable that CD73 is either a regulator or an effector of EGFR-EMT in HNSCC. EGFR activation by high-dose EGF may promote CD73 transcription, which may be required for proliferation, metabolism, and/or EGFR-EMT induction. Alternatively, CD73 may represent a target gene of EGFR-EMT with functional implications in local invasion (Fig. 4A). To test these hypotheses, local invasion was quantified after mitomycin C (MMC) treatment of FaDu cells at IC_50_ (0.02 µg/mL, 72 h). MMC treatment resulted in a generally reduced inhibition of viability and local invasion, but nonetheless inhibition of CD73 activity with 22E6 resulted in significant blocking of local invasion by 72% (Fig. 4B, C), suggesting a proliferation-independent effect of CD73 blockade on invasion.

Next, FaDu cells were treated with EGF^high^ in combination with 22E6, and morphologic and molecular signs of EMT were assessed. Blocking CD73 enzymatic function with 22E6 had no visible impact on morphologic changes observed upon EGF^high^ treatment. Lack of cell–cell adhesion and increased spindle-shape remained detectable after co-treatment with 22E6 (Fig. 4D). CDH1 (E-cadherin) mRNA levels were unchanged after EGF^high^ and EGF^high^ in combination with 22E6 compared to control. In contrast, EMT markers CDH2 (N-cadherin), TWIST1, and vimentin were significantly up-regulated after EGF^high^ treatment and no differences in induction was observed upon EGF^high^ and 22E6 co-treatments (Fig. 4E).

Since these results pointed at CD73 being an effector of EGFR-EMT, the specificity for CD73 in local invasion was initially assessed using CD73 inhibitor APCP. Treatment with APCP reduced EGF^high^-mediated local invasive area by 70.5% and comparably to 22E6 treatment (Fig. 4F). Next, we addressed gain- and loss-of-function of CD73 in local invasion. Knock-down of CD73 (CD73-KD) upon stable transfection with shRNA resulted in a complete loss of CD73, which was complemented by introduction of a CD73 expression plasmid in knock-down cells (KD-OE). Over-expression of CD73 (CD73-OE) was achieved in FaDu wild-type cells upon stable transfection of a CD73 expression plasmid (Fig. 4G, H). Local invasion of CD73-KD FaDu cells following treatment with EGF^high^ was significantly reduced compared to control cells (Fig. 4I, J, 39% reduction). Treatment with 22E6 remained functional in CD73-KD FaDu cells and further reduced local invasion to repressed levels observed in 22E6-treated control cells (Fig. 4I, J). Quantification of cell surface expression of CD73 revealed a re-expression of CD73 upon treatment with EGF^high^ to levels comparable to control cells under serum-free conditions (Fig. 4K). Thus, EGF^high^ treatment partly circumvented shRNA effects on CD73 expression, providing target molecules for the 22E6 antibody. Over-expression of CD73 in wild-type FaDu cells delivered a minor increase in local invasion (Fig. 4L, M) and entirely restored the capacity of CD73-KD cells to invade locally back to levels observed in wild-type FaDu cells (Fig. 4N, O). Hence, CD73 is a direct effector of EGFR-EMT in the cellular local invasion.

### CD73 is highly expressed in budding cells in HNSCC

**Fig. 5:**
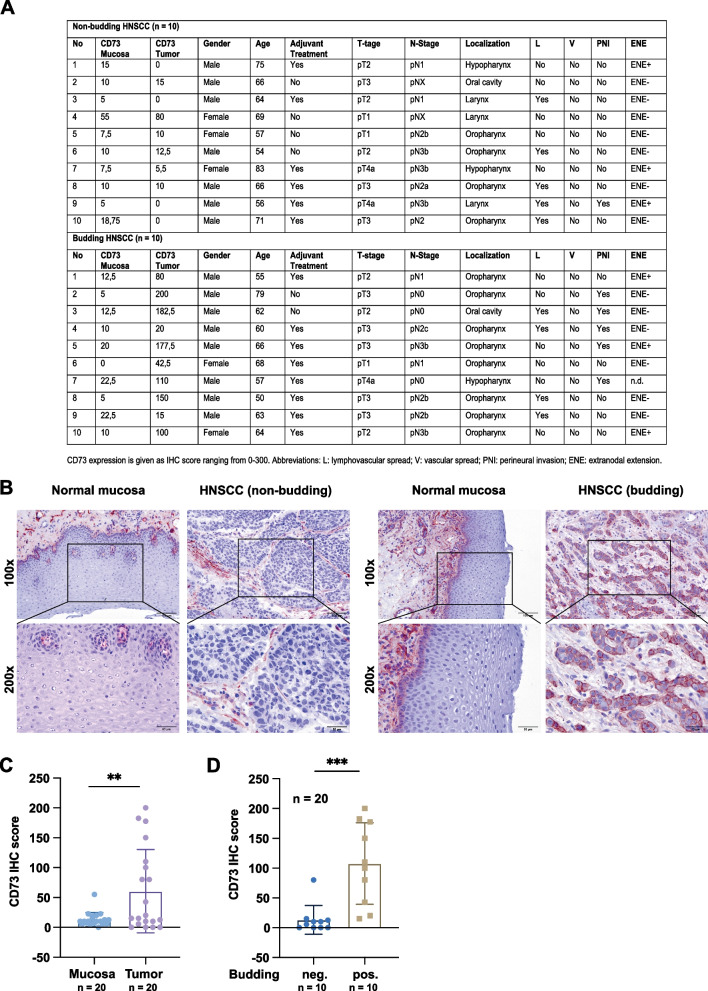
CD73 expression correlated with tumor budding in primary HNSCC. **A** Tabular depiction of non-budding and budding HNSCC (each n = 10) including CD73 IHC scores [34] in normal mucosa and primary HNSCC, gender, age, adjuvant treatment, T- and N-stage, localization, lymphatic and vascular infiltration, perineural invasion (PNI), and extranodal extension (ENE). **B** Representative CD73 IHC staining in normal mucosa and HNSCC from non-budding and budding tumors. Two magnifications are depicted with squares marking magnified areas. **C** Quantification of CD73 IHC scores in normal mucosa and primary tumors (n = 20 each) (left) and in non-budding (negative) and budding HNSCC (positive) (each n = 10). Shown are mean with SD. ** p < 0.01; *** p < 0.001

Tumor budding defines the detachment and local invasion of one to few cells, typically below five cells [35]. Based on the involvement of CD73 in local invasion in vitro, we next analyzed CD73 expression in normal mucosa and tumor samples of budding and non-budding HPV-negative HNSCC. Demographic parameters of each n = 10 patients with non-budding and budding HNSCC are presented in Fig. 5A. Tumor sizes and pathological/clinical parameters did not differ significantly across non-budding and budding samples, thus precluding effects on budding (Additional file 3: Table S1). Representative IHC staining of CD73 in normal mucosa and tumor are depicted in two magnifications in Fig. 5B. Quantification of CD73 using a scoring system implementing expression intensity (0–3) and proportion of positive cells (0–100%) [34] was conducted. Tumor samples showed a significantly elevated CD73 expression with a mean score of 60.53 versus 13.19 in normal mucosa and a difference between the means of 47.34 ± 15.77 (Fig. 5C and Additional file 3: Table S1). CD73 expression in epithelial cells of normal mucosa was predominant in the first suprabasal layer. Comparing CD73 expression in non-budding and budding HNSCC, a difference between the means of 94.45 ± 22.95 was observed (non-budding CD73 IHC score mean: 13.30; budding CD73 IHC score mean: 107.8) (Fig. 5D). Hence, CD73 expression tightly correlated with tumor budding in HNSCC samples and budding cells expressed CD73 strongly.

### CD73 correlation with EGFR activity, EMT, p-EMT, and EGFR-EMT in single malignant cells

**Fig. 6:**
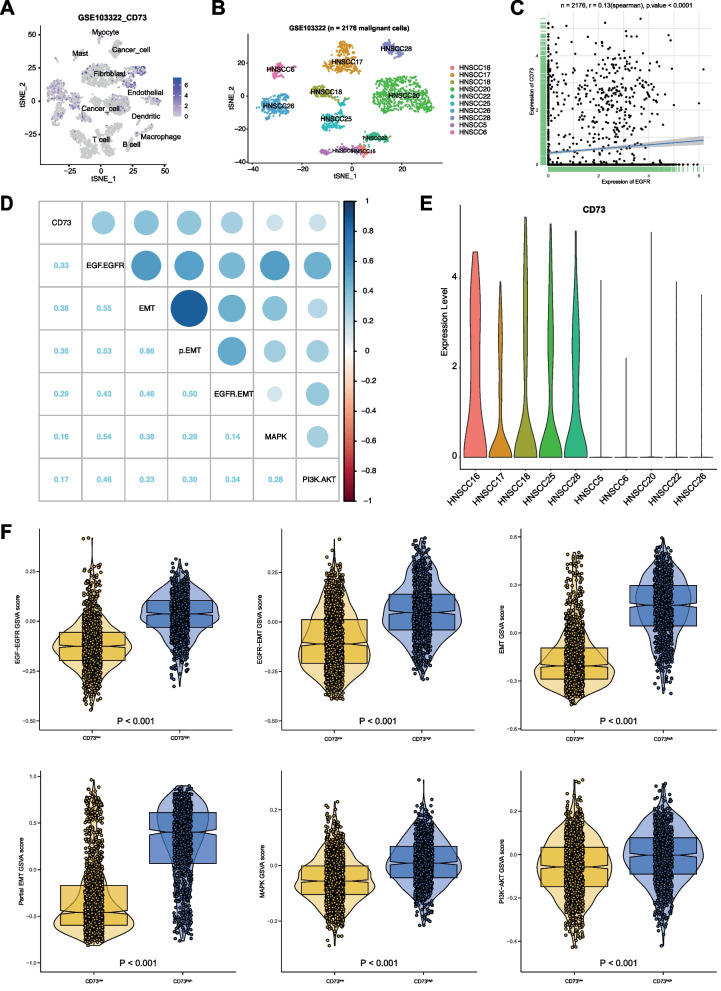
CD73 expression and correlation with EGFR activity, EMT, pEMT, and EGFR-EMT in scRNAseq of HNSCC. **A** A tSNE plot of the indicated major cell types identified in GSE103322 is depicted with the relative CD73 mRNA expression. **B** Malignant cells (n = 2716) are shown in a tSNE plot of n = 10 individual patients with highest single cell numbers from GSE103322. Patients´ capture is analogous to the original publication [44]. **C** CD73 and EGFR mRNA expression are shown from n = 2176 malignant cells of GSE103322 with Spearman correlation r-value and p-value. **D** Pearson correlation ρ values of CD73 mRNA expression and EGFR pathway activity, EGFR-EMT, hallmark EMT, p-EMT, MAPK and Akt activity GSVA scores across malignant cells (n = 2176) are shown in a correlation heat map for scRNAseq data from n = 10 patients from GSE103322. **E** CD73 mRNA expression across malignant cells (n = 2,176) is shown in violin plots for n = 10 patients from GSE103322. **F** EGFR pathway activity, EGFR-EMT, hallmark EMT, p-EMT MAPK and Akt activity GSVA scores across malignant cells (n = 2,176) from CD73^low^ and CD73^high^ patients are shown in violin plots for scRNAseq data from GSE103322

The expression of CD73 was assessed in single cell RNAseq (scRNAseq) dataset GSE103222, which comprises single cell transcriptomes from n = 18 oral cavity SCC [44]. CD73 expression was observed in malignant cells, fibroblasts, endothelial cells, and macrophages (Fig. 6A). Further analyses were restricted to malignant cells (Fig. 6B,  n = 2176 malignant cells from n = 10 HNSCC), which did not reveal a correlation between CD73 and EGFR gene expression (Spearman Rank correlation r = 01.3; Fig. 6C). Huang et al*.* reported a discrepancy between EGFR gene expression and activity in HNSCC patients, which was related to differing availability of EGFR ligands [14]. Therefore, global correlations of CD73 with EGFR activity, EMT subtypes, and MAPK and Akt activity were assessed in single malignant HNSCC cells. CD73 expression correlated with EGFR pathway activity, EGFR-EMT, hallmark EMT signature, and p-EMT. Spearman rank values for the correlation of CD73 with EGFR activity, hallmark EMT, p-EMT, and EGFR-EMT at the single cell level were 0.33, 0.38, 0.36, and 0.29, respectively. Correlations with MAPK and Akt activity were low despite being significant (Fig. 6D).

Patients were stratified in CD73^high^ and CD73^low^ groups of each five samples (Fig. 6E) and the distribution of scores of single cell gene set variation analyses (GSVA) of EGFR activity, EGFR-EMT, EMT, p-EMT, MAPK and Akt activity were compared between malignant cells of patient groups. CD73^high^ patients were characterized by high EMT, p-EMT, EGFR activity, and EGFR-EMT. Minorly enhanced MAPK activity and weak Akt activity association was seen in CD73^high^ HNSCC patients (Fig. 6F). Similarly, at the single patient level CD73^high^ HNSCC patients had comparably high GSVA scores for EMT, p-EMT, EGFR activity, and EGFR-EMT (Additional file 2: Fig. S2).

### CD73 expression in large cohorts of HPV-negative HNSCC

HPV-status is associated with significantly improved outcome and therefore is a confounding parameter when assessing prognostic values of genes [8]. High CD73 expression has been reported as a prognostic marker of poor survival in HNSCC [9, 36, 45, 60, 64]. However, HPV-status was not specified or accounted for in published data and hence survival curves have been computed in mixed HPV-negative and -positive tumor cohorts [36, 60, 64]. Importantly, Zhang et al*.* reported on a significantly enhanced CD73 expression in HPV-negative versus HPV-positive HNSCC (see their Fig. 2c), which strengthens a bias towards a correlation of higher CD73 expression with poorer clinical outcome. We have therefore re-addressed correlations of CD73 with parameters of interest and clinical endpoints in the HPV-negative TCGA-HNSCC (n = 415), the Fred Hutchinson Cancer Research (FHCRC) cohort of HPV-negative OSCC (n = 97; GSE41613), and HPV-negative HNSCC from the GSE65858 cohort (n = 196).

**Fig. 7:**
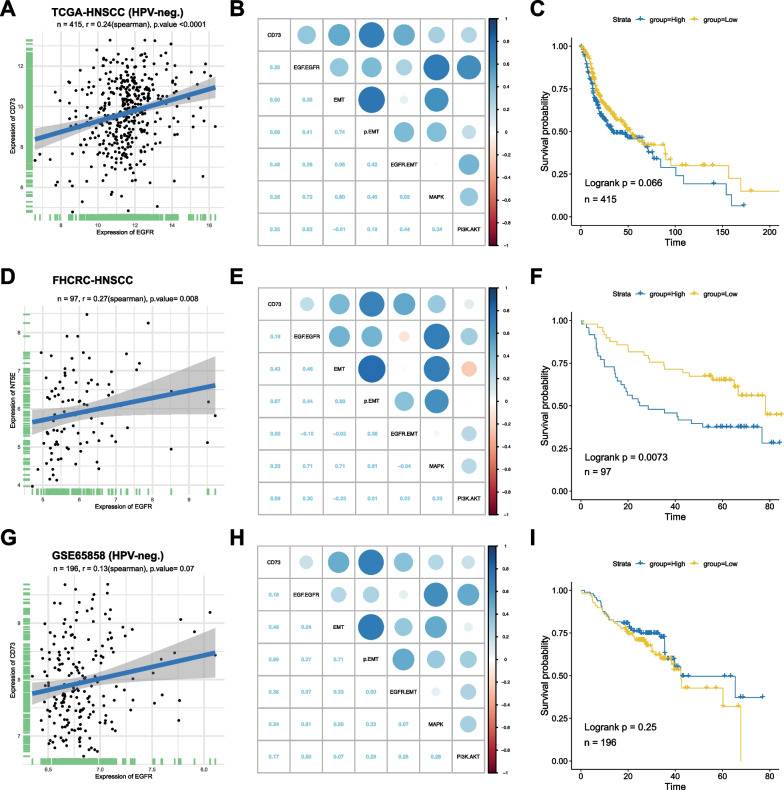
CD73 expression with overall survival in large clinical cohorts of HNSCC. **A**, **D**, **G** CD73 and EGFR mRNA expression are shown from bulk RNAseq datasets of HPV-negative HNSCC patients of TCGA (**A**), the Fred Hutchinson Cancer Research Center (**D**), and GSE65858 (**G**) with Spearman correlation r-values and p-values. **B**, **E**, **H** Pearson correlation ρ values of CD73 mRNA expression and EGFR pathway activity, EGFR-EMT, hallmark EMT, pEMT, MAPK and Akt activity GSVA scores across bulk RNAseq datasets of HPV-negative HNSCC patients of TCGA (**B**), the Fred Hutchinson Cancer Research Center (**E**), and GSE65858 (**H**). **C**, **F**, **I** Survival probabilities are depicted in Kaplan–Meier curves for HPV-negative HNSCC patients of TCGA (**C**; n = 415), the Fred Hutchinson Cancer Research Center (**F**; n = 97), and GSE65858 (**I**; n = 196) stratified for the median of CD73 expression with Logrank p-values

Correlations of CD73 expression with the EGFR gene expression, EGFR pathway activation and the EMT hallmark (both MSigDB signatures) [28, 50], the pEMT signature [44], and the EGFR-mediated EMT signature [48] were evaluated. In all three cohorts the correlation between the expression of CD73 and EGFR revealed low, with Spearman ρ values ranging from 0.13 to 0.27 (Fig. 7A, D, and G). Strongest correlations of CD73 expression were observed with the p-EMT signature (ρ value ranging from 0.67 to 0.69). Correlations with EGFR-mediated EMT ranged from ρ values of 0.38 to 0.53, correlations with MAPK activity from 0.24–0.33, and Akt activity from 0.09–0.25 (Fig. 7B, E, H).

Kaplan–Meier curves served to visualize associations of CD73 expression with OS in all three HPV-negative cohorts. As shown in Fig. 7C, F and I, CD73 showed a significant correlation with OS only in the FHCRC OSCC cohort but not in the TCGA and GSE65858 cohorts. Hence, CD73 correlated with OS only in OSCC but not across all HNSCC sub-localizations.

### CD73 expression as a predictive marker of Cetuximab response

**Fig. 8:**
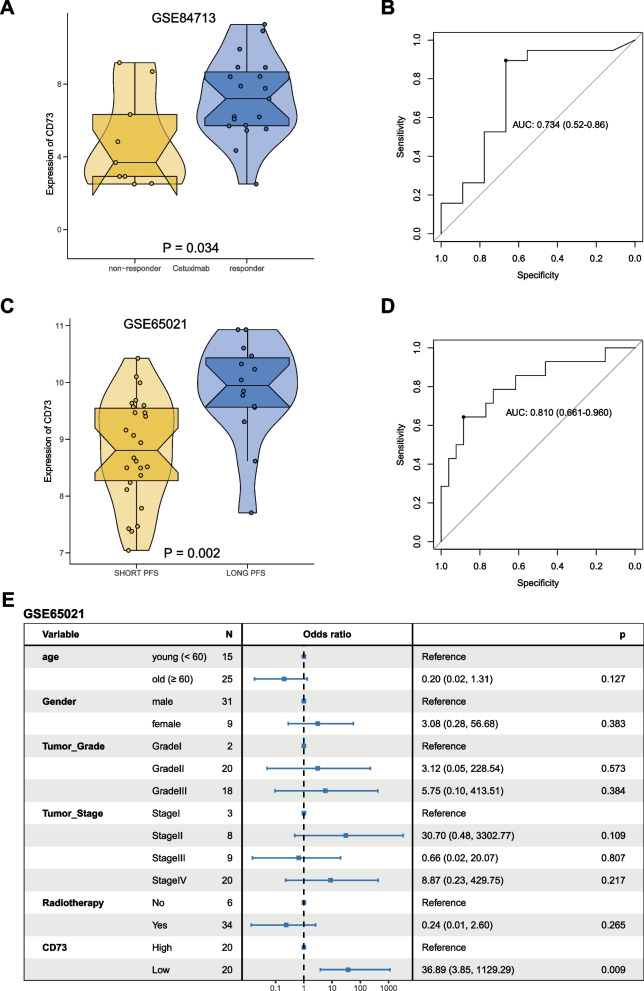
CD73 expression as an indicator of response to Cetuximab in HNSCC. **A** CD73 mRNA expression was analyzed in Cetuximab non-responder and responder PDX-derived HNSCC models within the GSE84713 dataset as violin plots with single data points and median values. **B** Receiver operating characteristics of specificity and sensitivity of the CD73 expression to distinguish non-responder and responder PDXs following Cetuximab treatment in xenotransplantation models (AUC: area under the curve). **C** CD73 mRNA expression was analyzed in the Bossi et al. cohort [5] comprised of advanced, metastasized HNSCC patients treated with Cetuximab with either short progression-free survival (PFS; n = 26; PFS < 3 months) or long PFS (n = 14; PFS > 19 months). CD73 mRNA expression is shown for short and long PFS as violin plot with single data points and median values. **D** Receiver operating characteristics of specificity and sensitivity of the CD73 expression to distinguish short and long PFS (AUC: area under the curve). **E** Multivariate linear regression model for the capacity of CD73 expression and clinical parameters (age, gender, tumor grade, tumor stage, and radiotherapy) to estimate the odds for short or long PFS. All variables are indicated including each reference, odds ratio, patient numbers (N), 95% CI, and p-value. CD73 was stratified according to median expression

The up-regulation of genes of the EGFR_EMT_Signature in patients may reflect a high degree of EGFR-EMT along with increased tumor cell dissemination and thus a benefit to treat patients with Cetuximab. Since CD73 is up-regulated as a gene of the EGFR_EMT_Signature, we reasoned that its expression might conversely predict response to Cetuximab. CD73 expression was analyzed in the GSE84713 dataset that contains gene expression profiles of n = 28 patient-derived xenotransplants (PDX; 26/28 HPV-negative) in dependency of Cetuximab treatment [23]. CD73 was significantly elevated in PDX that responded to Cetuximab treatment with a reduction of relative tumor volume after three weeks of treatment compared to non-responders (Fig. 8A). The AUC for specificity and sensitivity of CD73 to discriminate responders from non-responders was 0.734 (Fig. 8B).

In the following, CD73 expression was assessed in n = 40 advanced, metastasized HNSCC patients who underwent Cetuximab-based treatment [5]. Patients were selected based upon treatment response and comprised n = 26 short progression-free survival (PFS) (< 3 months) and n = 14 long PFS (> 19 months). CD73 expression was significantly higher in patients with high PFS and the AUC value or the discrimination of short versus long PFS was 0.810 (Fig. 8C, D). A multivariate linear regression model was computed for CD73 expression and clinical parameters (age, gender, tumor grade, tumor stage, and radiotherapy) to predict the odds to belong to either the short or long PFS group. Using a median split, patients with low CD73 expression were at significantly higher odds to belong to the group of short PFS. All other parameters were not significantly associated with PFS (Fig. 8E). Hence, CD73 is a promising candidate predictive gene for response to Cetuximab.

## Discussion

Advanced recurrent and metastatic HNSCC remain a huge unmet challenge for clinical treatment. Single cells or small clusters of malignant cells budding from the primary tumor prior to surgical intervention may escape resection and generate recurrences shortly after the pressure of adjuvant radio(chemo)therapy is released [1, 41]. It was suggested that EMT fosters a detachment and invasion of tumor cells to form buds of therapy-resistant cells [19], hence our interest in the role of EGFR-EMT in local invasion [48].

The rationale for CD73 as a target to inhibit local invasion was severalfold. Firstly, CD73 is part of the ‘EGFR-EMT_Signature’, which characterizes migratory, invasive cells following sustained EGF treatment, and might hence be involved in the regulation of local invasion [48]. Secondly, CD73 is extracellularly accessible, which facilitates blocking of its enzymatic activity. For this purpose, we made use of the recently described antagonizing 22E6 antibody [21]. 22E6 specifically blocks membrane-tethered CD73 but not soluble CD73, and hence might have a higher specificity for tumor cells that express high levels of membrane CD73 [21]. Thirdly, CD73 has been implicated in central aspects of tumor progression including the suppression of immune cells via ADO production, the regulation of proliferation, migration, angiogenesis, EMT, and a potential involvement in metastasis formation in various cancers including HNSCC [6, 10, 12, 18, 26, 29–33, 37, 52, 55, 56, 59, 60, 62, 63, 65]. Currently available literature showed a function of CD73 in the regulation of EGFR phosphorylation through c-src and in the activation of the MAPK cascade [45, 60]. However, the reverse scenario of CD73 activation through EGFR signaling has not been studied and was therefore the subject of the present work.

Blocking the function of CD73 via 22E6 significantly suppressed migration and invasion induced by EGF treatment independently of proliferation effects. This finding is in line with reports of a detrimental effect of CD73 knockdown in Kyse30 and TE1 esophageal carcinoma cell lines [10] and an up-regulation of CD73 in lymph-homing breast cancer cells and in derived metastases in orthotopic models [26, 56]. Inhibitory effects of 22E6 on EGFR-mediated local invasion into ECM were demonstrated in a spheroid 3D model (Fig. 3A, B). Importantly, combining concentrations of Cetuximab and 22E6 that were per se inefficient resulted in very potent inhibition of the invasive area and in substantial reduction of the invasive distance of FaDu cells. In fact, CD73 inhibition with 22E6 concentrations that do not affect local invasion led to a substantial decrease in the functional IC_50_ of Cetuximab, pointing at complementary functions of EGFR and CD73 in the regulation of local invasion in HNSCC (Fig. 3C–G).

Effects of 22E6 were dependent upon CD73 expression as demonstrated with loss- and gain-of function cell variants (Fig. 4). The relevance of CD73 was further demonstrated in clinical samples, where CD73 expression was positively correlated with the degree of tumor budding. CD73 was over-expressed in primary tumors compared to matched normal mucosa and its expression was significantly enhanced in primary HNSCC characterized by strong budding. Importantly, single budding cells revealed strongly positive for CD73, further qualifying CD73 as a target molecule on these problematic tumor cells (Fig. 5). Hence, CD73 is an effector molecule in tumor cell migration and invasion in vitro and correlates with local dissemination in situ in primary human HNSCC. Based on the possibility to block CD73´s enzymatic function and tumor cell invasion, we propose that CD73 represents an attractive target molecule for adjuvant treatment of HNSCC in co-treatment regimens with Cetuximab. Dual targeting involving CD73 is functional and has been reported. For example, targeting CD73 and adenosinergic/purinergic pathways is supported by several approaches to boost radiotherapy effects [2], to circumvent ADO-dependent immunosuppression [42, 51], and for immune checkpoint modulation [58, 61]. The newly described function of CD73 downstream of EGFR is particularly attractive regarding options to overcome resistance to EGFR-targeted therapy [25, 38].

Non-immunological roles of CD73 progression have been postulated, including a crosstalk with EMT and a correlation between CD73 expression and the EMT score, amongst other cancers, in HNSCC [12, 18, 31, 40]. We have critically expanded these findings and show that CD73 correlated specifically with EGFR activity, EGFR-EMT, and p-EMT in addition to EMT. Since p-EMT and subsets of EGFR-EMT genes are linked to metastases formation, unfavorable clinical parameters, and reduced survival [44, 47, 48, 53], these novel associations strengthen the importance of CD73 in HNSCC progression as a mediator of EGFR functions. In HNSCC, CD73 was reported to the MAP-kinase pathway, invadopodia formation, and lung metastases formation in a rodent tumor model [60]. EGFR-EMT is primarily induced via the MEK-ERK1/2 pathway [39, 48], which suggested a possible positive-feedback loop between EGFR and CD73 in regulating EMT in HNSCC that converges at the MAPK level. It must be noted though that blocking CD73 function had no measurable effect on EGFR-EMT induction, thus qualifying CD73 as an effector rather than a regulator of EGFR-EMT in the cellular systems analyzed in the present study. Ultimately, Cetuximab- and 22E6-based inhibition of CD73 expression and function, respectively, may primarily target malignant cells undergoing EGFR-EMT and thus potentially aggressive cells [48].

High expression of CD73 has been linked to poorer OS and DFS in HNSCC patients [9, 30, 45, 60, 64]. However, in these reports, the HPV-status was disregarded when analyzing the association of CD73 with clinical endpoints, despite noting that HPV infection and CD73 were correlated [9]. HPV-positive HNSCC have an improved clinical outcome [8, 57] and a lower median CD73 expression (Fig. 2 in [64]), which represents a confounder when analyzing prognostic values of CD73. Here, we have modelled prognostic values of CD73 exclusively in HPV-negative patients of the TCGA-HNSCC, the Fred Hutchinson Cancer Research Center (FHCRC), and the GSE6585 cohorts. Although others published a prognostic value for CD73 in predicting OS and DFS in the TCGA-HNSCC cohort, we did not observe any significant difference in n = 415 HPV-negative HNSCC patients with respect to CD73 expression levels (Fig. 7A–C). Patient numbers indicated in previous publications suggested the implementation of the full TCGA cohort comprised of HPV-negative and -positive patients [9, 60, 64], which could account for the divergent results presented here. We observed a positive, significant correlation of high-level CD73 with poorer OS in the OSCC FHCRC cohort but not in the GSE65858 cohort (Fig. 7I). The FHCRC cohort is composed of oral cancers and the finding of a correlation of CD73 with clinical endpoints is thus in line with reports on the OSCC-JKLOD cohort [60]. We thus deduce that CD73 could be a marker for OSCC but not for other localizations of HPV-negative HNSCC. It must however be noted that unlike the large TCGA cohort, the JKLOD and the FHCRC cohorts are comprised of fewer cases (n = 122 and n = 97, respectively), which may affect correlations and significances.

The value of CD73 expression in primary tumors as surrogate marker for the response to Cetuximab was tested in Cetuximab-treated PDX [23] and a cohort of advanced, metastatic HNSCC [5]. PDX responding to Cetuximab were associated with a basal molecular subtype that is characterized by hypoxia and the expression of components of the EGFR pathway [20]. We observed that “responder” PDX samples expressed significantly higher levels of CD73 (Fig. 8A). Similarly, Cetuximab-treated patients with long PFS (> 19 months) showed significantly higher CD73 expression than patients with short PFS (< 3 months) (Fig. 8C). In this cohort, low expression of CD73 was associated with a higher odd for short PFS, suggesting that CD73 predicts response to Cetuximab treatment in advanced stage HNSCC (Fig. 8E). This finding is interesting since the mere expression of EGFR is not predictive of Cetuximab sensitivity. Hence, surrogate markers of EGFR-related aggressiveness of malignant cells may help to define patients who could benefit from EGFR blockade. CD73 and ITGB4 expression, as was shown recently by our group [48], may reflect the EGFR-EMT status of cells and thereby represent a sensor for detrimental, invasive malignant cells that react to Cetuximab.

Limitations of the present study can be seen in the restricted number of cell lines used for functional characterization of the effects of 22E6 on local invasion. However, effects were highly comparable and observed in 2D and 3D models. Furthermore, cohorts of Cetuximab-treated patients remain small, which may impair the presented analysis, despite statistical relevance.

## Conclusions

In summary, the 5ʹ-nucleotidase CD73/NT5E was characterized as a gene of the ‘EGFR-EMT_Signature’ involved in the regulation of local invasion. The 22E6 antibody is a novel option for targeting locally invading cells, potentially in combination with Cetuximab, and CD73 may serve as predictive marker for the response to Cetuximab treatment in advanced HNSCC patients.

### Supplementary Information

**Additional file 1: Figure S1**. Cytotoxicity of 22E6 antibody in FaDu and Kyse30 cell lines. FaDu (A) and Kyse30 (B) cells were treated with the indicated concentrations of anti-CD73 22E6 antibody. Cell viability was assessed via WST8 measurements at 24 h (left panels) and 72 h (right panels). Shown are mean with SD from n = 3 independent experiments performed in triplicates. Ns: not significant.

**Additional file 2: Figure S2. **EGF activity, EGFR-EMT, EMT, p-EMT, MAPK and PI3K-Akt activity scores. Gene set variation analysis scores of EGF activity, EGFR-EMT, EMT, p-EMT, MAPK and PI3K-Akt activity were calculated for n = 2,176 malignant cells with n = 10 HPV-negative HNSCC patients within GSE103322. GSVA scores are depicted as violin plots for each patient individually. Patients 1–5 are CD73^high^, patients 6–10 are CD7^3low^.

**Additional file 3: Table S1. **

## Data Availability

All data generated or analyzed during this study are included in this published article. Publicly available bulk and single cell RNA sequencing datasets analyzed in the present work are listed with GSE numbers in the section “Dataset source and preprocessing”.

## Abbreviations

2D: Two-dimensional
3D: Three-dimensional
AUC: Area under the curve
ADO: Adenosine
AMP: Adenosine monophosphate
CAF: Cancer-associated fibroblast
EGF: Epidermal growth factor
EGFR: Epidermal growth factor receptor
EMT: Epithelial-to-mesenchymal transition
GSE: Genomic spatial event database
GSVA: Gene set variation analysis
HNSCC: Head and neck squamous cell carcinoma
KD: Knock-down
Log2FC: Log2 fold change
LK: Lymph node
LR: Local recurrence
MRD: Minimal residual disease
MMC: Mitomycin C
NT5E: 5´-Nucleotidase Ecto
OE: Over-expression
OS: Overall survival
OSCC: Oral squamous cell carcinoma
PDX: Patient-derived xenotransplant
PFS: Progression-free survival
p-EMT: Partial epithelial-to-mesenchymal transition
scRNAseq: Single cell RNA sequencing
