# Supplementary figures and images for: 5ʹ-Ectonucleotidase CD73/NT5E supports EGFR-mediated invasion of HPV-negative head and neck carcinoma cells
Source: J Biomed Sci. 2023 Aug 24;30:72. doi: 10.1186/s12929-023-00968-6 (PMC10463398; doi:10.1186/s12929-023-00968-6)

**WST8\_24h\_Fadu**

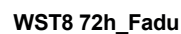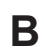

**WST8\_24h\_Kyse30**

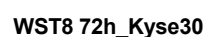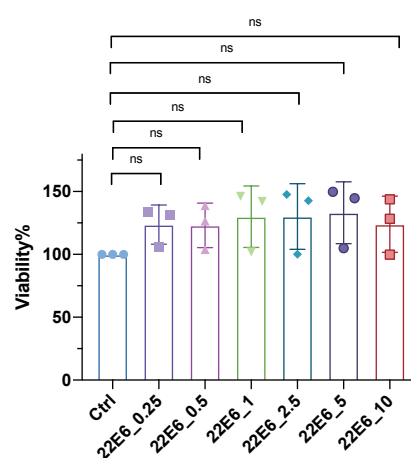

Supplement: Supplementary file 1 — Additional file 1: Figure S1. Cytotoxicity of 22E6 antibody in FaDu and Kyse30 cell lines. FaDu (A) and Kyse30 (B) cells were treated with the indicated concentrations of anti-CD73 22E6 antibody. Cell viability was assessed via WST8 measurements at 24 h (left panels) and 72 h (right panels). Shown are mean with SD from n = 3 independent experiments performed in triplicates. Ns: not significant. [file 12929_2023_968_MOESM1_ESM.pdf]

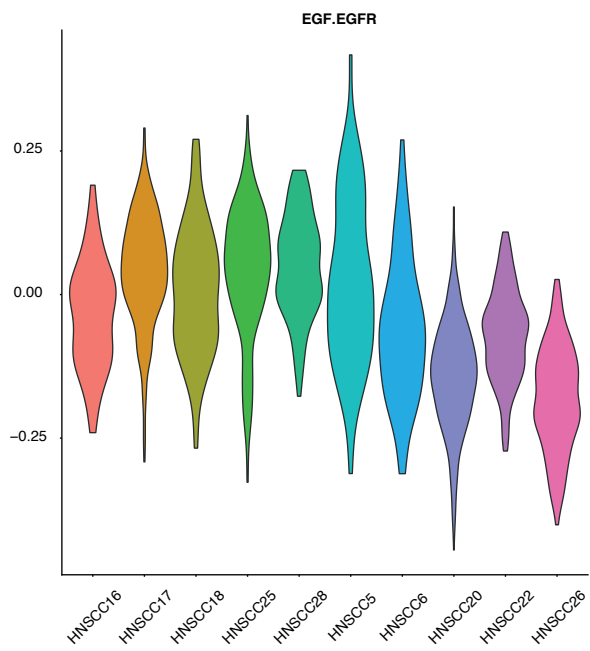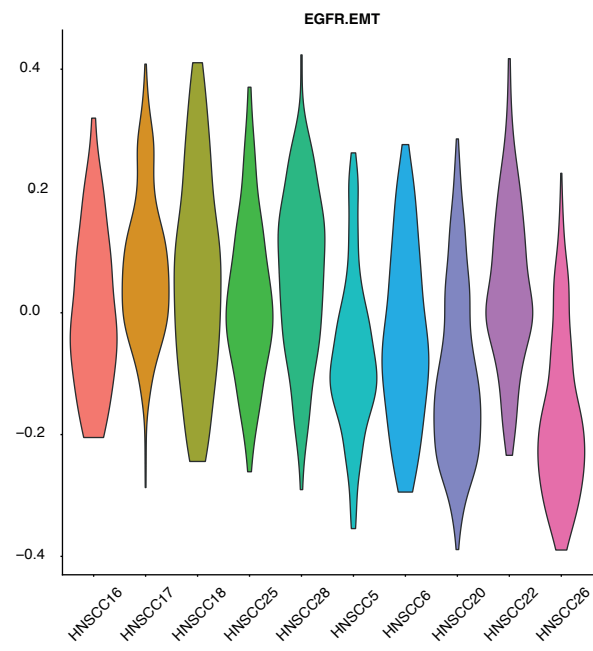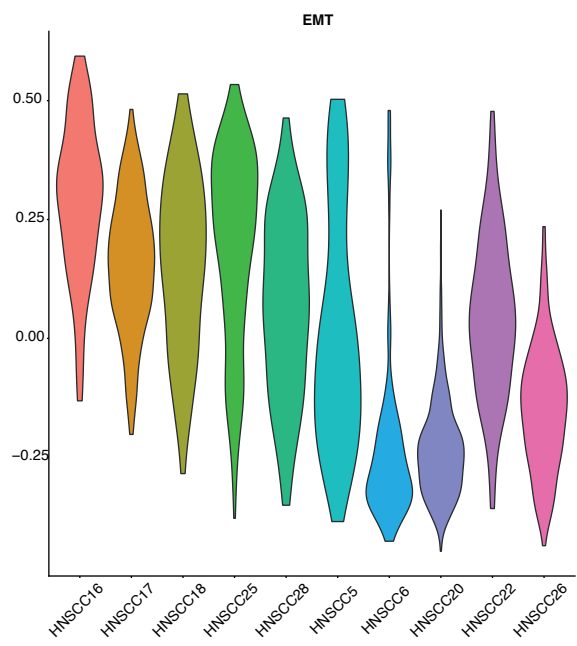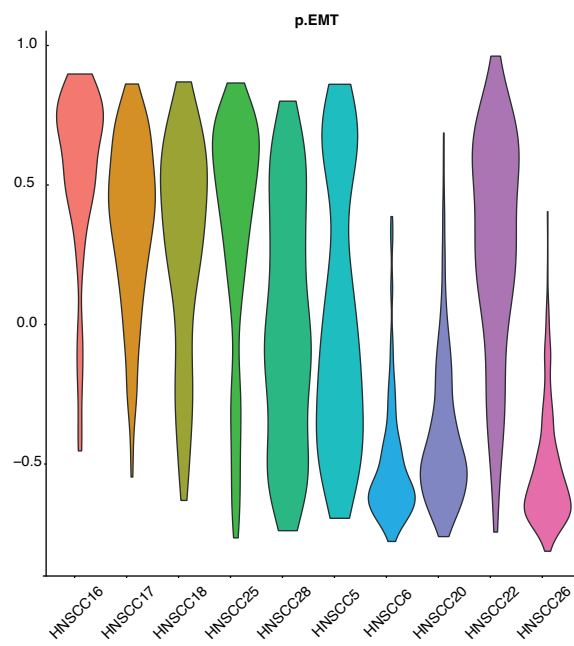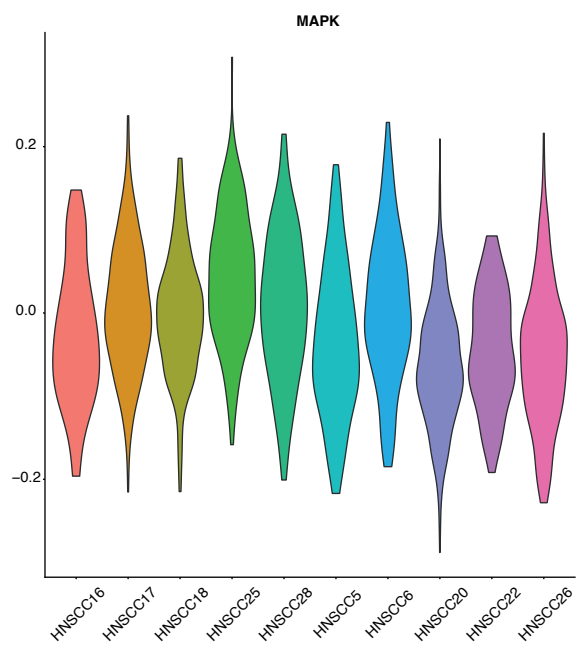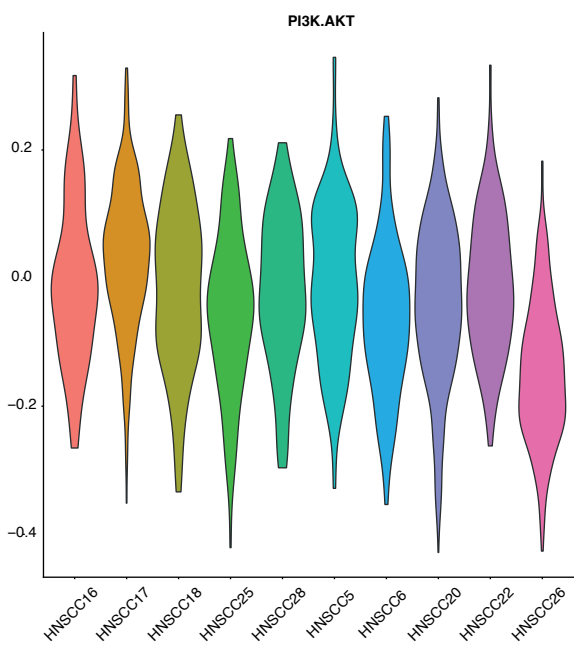

Supplement: Supplementary file 2 — Additional file 2: Figure S2. EGF activity, EGFR-EMT, EMT, p-EMT, MAPK and PI3K-Akt activity scores. Gene set variation analysis scores of EGF activity, EGFR-EMT, EMT, p-EMT, MAPK and PI3K-Akt activity were calculated for n = 2,176 malignant cells with n = 10 HPV-negative HNSCC patients within GSE103322. GSVA scores are depicted as violin plots for each patient individually. Patients 1–5 are CD73high, patients 6–10 are CD73low. [file 12929_2023_968_MOESM2_ESM.pdf]
