# Supplementary material for: 5ʹ-Ectonucleotidase CD73/NT5E supports EGFR-mediated invasion of HPV-negative head and neck carcinoma cells
Source: J Biomed Sci. 2023 Aug 24;30:72. doi: 10.1186/s12929-023-00968-6 (PMC10463398; doi:10.1186/s12929-023-00968-6)
Supplement: Supplementary file 3 — Additional file 3: Table S1. [file 12929_2023_968_MOESM3_ESM.docx]

**Supplementary Table 1: Clinical parameters of budding and non-budding samples of HNSCC**

| **Clinical parameters** | **Budding (n=10)** | **Non-budding(n=10)** | **p-value** |
| --- | --- | --- | --- |
| **Gender** |  |  |  |
| Female  male  **Adjuvant treatment**  Yes  No  Unknown  **T-stage**  T1  T2  T3  T4a  **N-stage**  N0  N1  N2  N3  Nx  **N-Status**  N+  N-  Unknown  **Localization**  Hypopharynx  Oral  Oropharynx  larynx  **Grading**  G2  G3  Unknown  **Lymphovascular invasion**  Yes  No  **Angioinvasion**  No  **Perineural invasion**  Yes  No  **ENE**  ENE+  ENE-  Unknown  **Smoking status**  Current  Former  Never  **CD73_Tumor**  High (score＞17.5)  Low (score≤17.5) | 2  8  8  2  0  1  3  5  1  3  2  3  2  0  7  3  0  1  3  6  0  2  8  0  4  6  10  5  5  3  6  1  4  6  0  9  1 | 3  7  6  3  1  2  3  3  2  0  2  3  3  2  8  1  1  1  1  4  4  0  9  1  5  5  10  1  9  3  7  0  6  3  1  1  9 | 1  0.476  0.761  0.267  0.356  0.145  0.217  1  1  0.143  0.584  0.301  0.002 |
